# Hemofiltration with the Seraph^®^ 100 Microbind^®^ Affinity filter decreases SARS-CoV-2 nucleocapsid protein in critically ill COVID-19 patients

**DOI:** 10.1186/s13054-021-03597-3

**Published:** 2021-06-01

**Authors:** Jan T. Kielstein, Dan-Nicolae Borchina, Thomas Fühner, Soyoon Hwang, Dawn Mattoon, Andrew J. Ball

**Affiliations:** 1 Medical Clinic V, Nephrology | Rheumatology | Blood Purification, Academic Teaching Hospital Braunschweig, Salzdahlumer Strasse 90, 38126 Braunschweig, Germany; 2 Department of Respiratory Medicine, Hannover Medical School, 30623 Hannover, Germany; 3 Quanterix Corporation, Billerica, MA 01821 USA

The nucleocapsid protein (N-protein) of SARS-CoV-2 is a structural protein that oligomerizes to form a complex surrounding viral RNA, thus protecting it from the host cell environment. It is abundantly expressed within infected cells, where it facilitates viral RNA transcription, an essential step for viral replication Recently an ultrasensitive Simoa^®^ immunoassay has been described that robustly measures SARS-CoV-2 N-protein in venous blood, dried blood microsamples, and saliva [1]. This study measured N-protein in longitudinal blood samples of COVID-19 patients and demonstrated readily detectable viral antigen two weeks after initial positive PCR testing, with concentrations gradually decreasing, inversely correlated with anti-SARS-CoV2 adaptive immune response. This study supports observations reported elsewhere that viral load in blood correlates with disease severity [2].

The Seraph^®^ 100 Microbind^®^ Affinity adsorber (Exthera Medical, CA, USA) is an extracorporeal treatment currently being explored as an approach to improve the clinical course and outcome of critically ill patients with COVID-19. On April 17, 2020, the FDA granted emergency use authorization for the Seraph^®^ 100 for use in the context of severe and critical disease, for which effective treatment options are limited. Bacteria and viruses bind to the immobilized heparin on the ultra-high molecular weight polyethylene beads of the Seraph^®^ device in a manner similar to the interaction with heparan sulfate on the cell surface and are thereby removed from the bloodstream [3]. The spike protein of SARS-CoV-2 has been shown to bind to cellular heparan sulfate (and heparin) through its receptor-binding domain, and recent studies suggest the heparin binding of the spike protein is much more pronounced in SARS-CoV-2 than in other coronaviruses [4]. In addition to an anecdotal report [5] a recent multicenter study showed that mortality of COVID-19 patients was much lower (37.7%) in the Seraph 100 treated group compared to a control group (67.4%) [6].

Here, we report the effect of the Seraph treatment on the concentration of the N-protein in critically ill COVID-19 patients as part of an ongoing biomarker study, approved by the IRB of the Hannover Medical School (9130_MPG_23b_2020). Six out of seven COVID-19 patients exhibited measurable concentrations of the N-protein prior to treatment with the Seraph^®^ device, that seemed to be related to the severity of the disease and the duration of the disease (Table 1). While hemoperfusion with the Seraph^®^ was executed either alone or in combination with a wide range of supportive treatments, including intermittent hemodialysis and continuous renal replacement therapy, N-protein concentration was consistently reduced when comparing pre- and post- Seraph treatment blood samples (Table 1). Calculating the Seraph whole blood clearance (CL) by the nucleocapsid concentration upstream (*C*_in_) and downstream (*C*_out_) of the Seraph and the blood flow (*Q*_B_) by the formula: CL = (*C*_in_ / *C*_out_) / *C*_in_ × *Q*_B_, resulted in a measurable device clearance that was not observed with other proteins including total serum protein (Fig. 1).

**Table 1:** Patient characteristics (laboratory data obtained on the day of Seraph^®^ treatment)

	# 1	# 2	# 3	# 4	# 5	# 6	# 7
Gender	M	F	M	M	F	M	M
Age (years	77	78	54	41	61	65	72
Weight (kg)	76.5	75	107	119	122	60.5	71
Height (cm)	164	160	188	180	168	166	175
Onset of symptoms-Seraph treatment (d)	5	19	10	13	9	16	12
Died @ hospital day	60	9	7	Survivor	Survivor	Survivor	18
N-protein before Seraph therapy (pg/mL)	1021.3	26.6	121,884.2	1070.2	1036.5	19.5	-
N-protein after Seraph therapy (pg/mL)	769.6	24.1	111,035.2	931.5	376.2	12.1	-
CRP (mg/l)	73	443	87	110	156	129	39
Ferritin (ng/mL)	2957	830	10,005	838	589	756	1,412
PCT (µg/L)	0.7	18.5	4.4	1.1	0.1	0.1	0.1
D-dimer (mg/L)	4.68	35.2	3.26	1.04	0.90	4.00	35.2
Therapy (h)	IHD (4)	CRRT (24)	IHD (4)	IHD (5)	HP (15)	HP (4)	HP (14)
Qb (mL/min)	300	90	250	250	80	200	100

*IHD* intermittent hemodialysis, *CRRT* continuous renal replacement therapy, *HP* hemoperfusion, *Q*_b_ blood flow

**Fig. 1:**
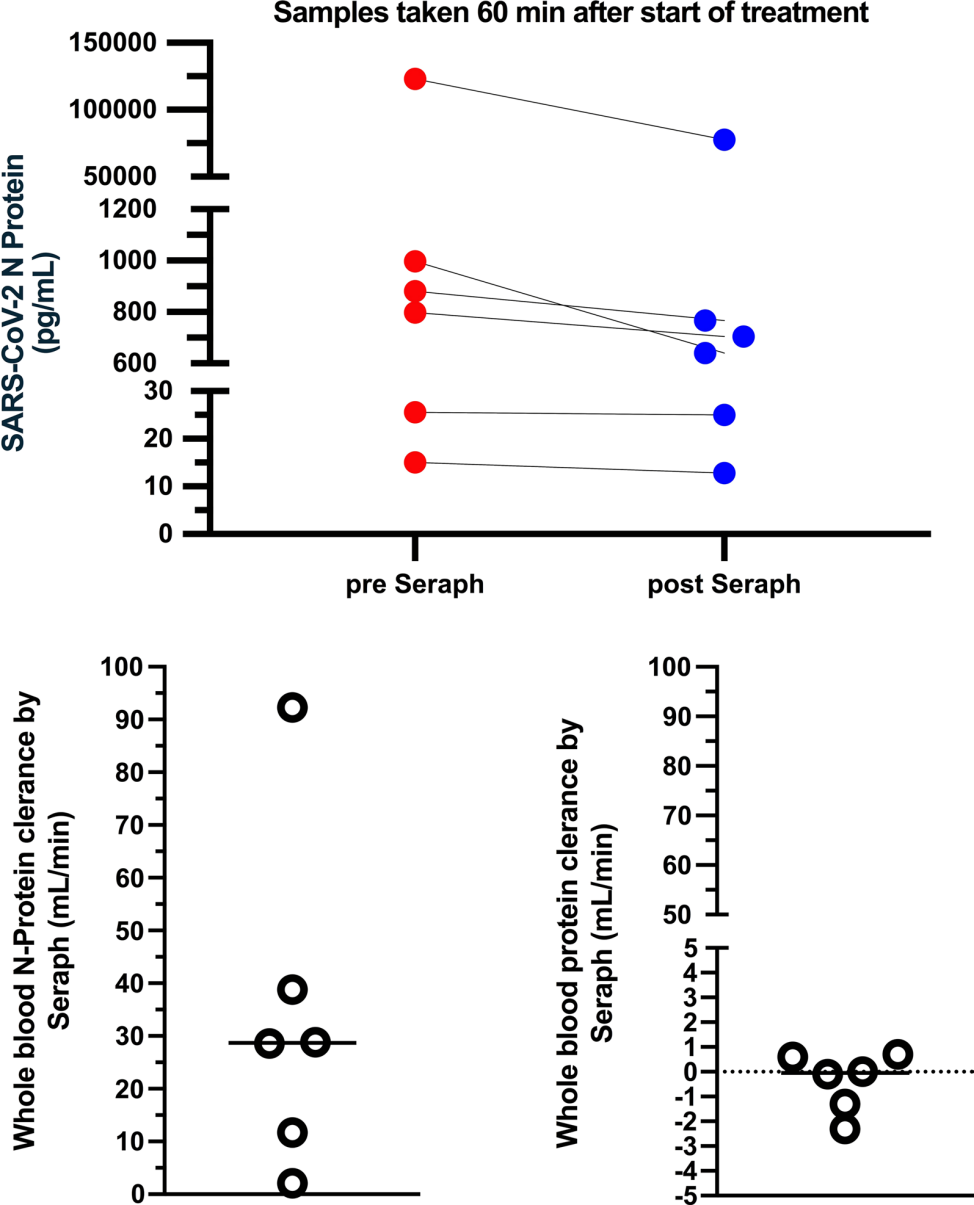
Concentration of the nucleocapsid protein pre (upstream) and post (downstream) of the Seraph^®^ at 60 min of treatment (upper part of the figure) and the resulting device clearance for the N-protein as well as the total plasma protein (lower part of the figure)

In conclusion, treating critically ill COVID-19 patients with the Seraph^®^ 100 Microbind^®^ Affinity filter decreased SARS-CoV-2 nucleocapsid protein in blood. The effect of clinically relevant outcome parameters needs to be determined.

## Data Availability

The datasets during and/or analyzed during the current study are available from the corresponding author on reasonable request.
